# Supplementary figures and images for: Arginase 1 is a key driver of immune suppression in pancreatic cancer
Source: eLife. 2023 Feb 2;12:e80721. doi: 10.7554/eLife.80721 (PMC10260021; doi:10.7554/eLife.80721)

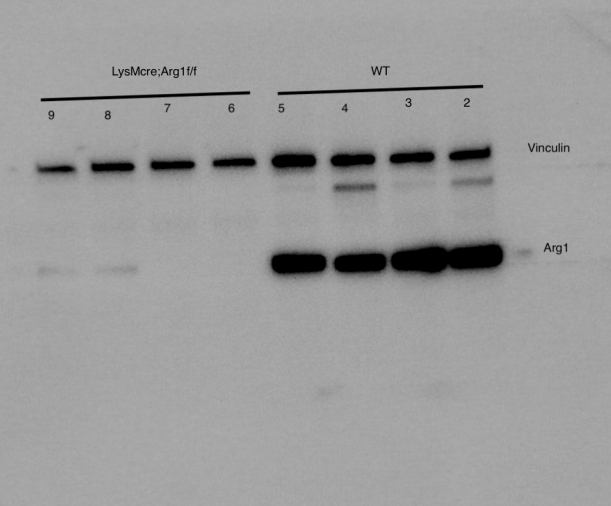

Supplement: Figure 2—source data 1. — Vinculin is the loading control, 124 kDa. ARG1 is 40 kDa. [file elife-80721-fig2-data1.zip › Fig2-source data/Fig2-sourceData1b.pdf]

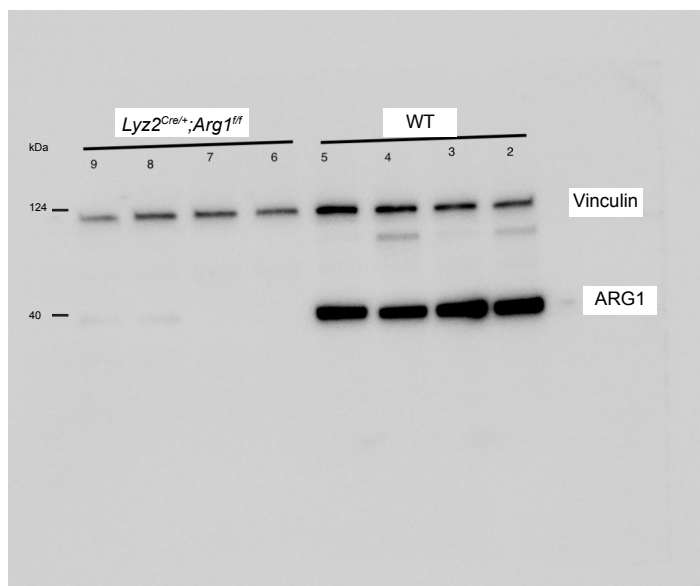

Supplement: Figure 2—source data 1. — Vinculin is the loading control, 124 kDa. ARG1 is 40 kDa. [file elife-80721-fig2-data1.zip › Fig2-source data/Fig2-sourceData1a.pdf]
